# Decreased SLC27A5 Suppresses Lipid Synthesis and Tyrosine Metabolism to Activate the Cell Cycle in Hepatocellular Carcinoma

**DOI:** 10.3390/biomedicines10020234

**Published:** 2022-01-22

**Authors:** Jiyan Wang, Yaya Qiao, Huanran Sun, Hongkai Chang, Huifang Zhao, Shuai Zhang, Changliang Shan

**Affiliations:** 1 State Key Laboratory of Medicinal Chemical Biology, College of Pharmacy and Tianjin Key Laboratory of Molecular Drug Research, Nankai University, Tianjin 300350, China; wangjy@mail.nankai.edu.cn (J.W.); 2120191147@mail.nankai.edu.cn (Y.Q.); 2120201192@mail.nankai.edu.cn (H.S.); 2120201229@mail.nankai.edu.cn (H.C.); 2 School of Integrative Medicine, Tianjin University of Traditional Chinese Medicine, Tianjin 301617, China; ZHF3920@163.com; 3 State Key Laboratory of Drug Research, Shanghai Institute of Materia Medica, Chinese Academy of Sciences, Shanghai 201203, China

**Keywords:** hepatocellular carcinoma, solute carrier family 27 member 5 (SLC27A5), tyrosine metabolism, lipids synthesis, cell cycle

## Abstract

Tyrosine is an essential ketogenic and glycogenic amino acid for the human body, which means that tyrosine is not only involved in protein metabolism, but also participates in the metabolism of lipids and carbohydrates. The liver is an important place for metabolism of lipids, carbohydrates, and proteins. The metabolic process of biological macro-molecules is a basis for maintaining the physiological activities of organisms, but the cross-linking mechanism of these processes is still unclear. Here, we found that the tyrosine-metabolizing enzymes, which were specifically and highly expressed in the liver, were significantly down-regulated in hepatocellular carcinoma (HCC), and had a correlation with a poor prognosis of HCC patients. Further analysis found that the reduction of tyrosine metabolism would activate the cell cycle and promote cell proliferation. In addition, we also found that the solute carrier family 27 member 5 (SLC27A5) regulates the expression of tyrosine-metabolizing enzymes through nuclear factor erythroid 2-related factor 2 (NRF2). Therefore, the SLC27A5 and tyrosine-metabolizing enzymes that we have identified coordinate lipid and tyrosine metabolism, regulate the cell cycle, and are potential targets for cancer treatment.

## 1. Introduction

The amino acid is the constituent unit of protein, and its important physiological function is to participate in protein synthesis as raw materials [1]. The protein is in a dynamic balance of continuous synthesis and decomposition; therefore, amino acid metabolism is the central content of protein catabolism. About 1–2% of the protein in the adult body is degraded every day, and about 70–80% of the amino acids produced by protein degradation are reused to synthesize new proteins, and the rest are converted into other substances. Fumarate and acetoacetate, produced by tyrosine metabolism, transform acetyl-CoA through glucose and lipid metabolism, respectively [2]. Therefore, the abnormality of amino acid metabolic pathways will change the metabolic flux of other metabolic pathways, and ultimately lead to the occurrence of diseases. For example, we previously found that 4-hydroxyphenylpyruvate dioxygenase (HPD), as a tyrosine-metabolizing enzyme, regulates the pentose phosphate pathway (PPP) flux to promote the growth of lung cancer [3]. Besides, Li et al. found that overexpression of glutathione S-transferase zeta-1 (GSTZ1) significantly decreased the glycolytic rate of hepatoma cells, whereas knockdown GSTZ1 had the opposite effect [4]. More importantly, loss of fumarylacetoacetate hydrolase (FAH) causes severe hepatocyte damage [5]. These indicate that the tyrosine metabolic pathway has important connections to other metabolic pathways of cells and individuals.

The liver is the largest gland and a substantial organ in the human body, and its unique structural characteristics endow the liver with complex and diverse biochemical functions. The liver not only plays a central role in the metabolism of glucose, lipids, and proteins, but also has various physiological functions such as biotransformation, secretion and excretion [6]. These metabolic processes are not independent, but closely linked, coordinated, and interacted. It is generally believed that the mechanistic target of the rapamycin (mTOR) pathway integrates nutritional signaling pathways (glucose and amino acids) to synthesize biological macromolecules (lipids, proteins and nucleotides) [7,8]. However, there is no clear understanding of the direct cross-linking of metabolic processes in hepatocellular carcinoma (HCC).

The solute carrier family 27 member 5 gene (*SLC27A5*), a member of the SLC27A gene family, encodes fatty acid transport protein 5 (FATP5), which is highly expressed in the liver. It functions in fatty acid transport and bile acid metabolism [9], which is an important risk factor for nonalcoholic fatty liver disease (NAFLD) [10,11]. The uptake of external fatty acid is significantly decreased in SLC27A5-knockout mice, while de novo synthesis is increased in hepatocytes, and their weight could not increase even under a high-fat diet [12,13]. In addition, SLC27A5 is also a potential prognostic biomarker for hepatocellular carcinoma [14]. However, the relationship between SLC27A5 and other metabolic pathways is unclear in HCC.

Here, we found that tyrosine-metabolizing enzymes are significantly down-regulated in HCC, and abnormally activate the cell cycle to promote cell proliferation. Meanwhile, we also found that SLC27A5 is an upstream regulator that promotes the expression of tyrosine-metabolizing enzymes, and the regulation process depends on the nuclear factor erythroid 2-related factor 2 (NRF2) factor. In HCC patients, the expression of SLC27A5 is significantly reduced, and the transport of free fatty acids to liver tissue is reduced, which inhibits the patient’s uptake of exogenous fatty acids. Meanwhile, it also inhibits the expression of tyrosine-metabolizing enzymes, resulting in the hindrance of endogenous lipid synthesis, which explains the weight loss of HCC patients. Collectively, our results implicate SLC27A5 and tyrosine-metabolizing enzymes as acting as tumor suppressor in HCC and coordinating lipid and tyrosine metabolism, which may be potential targets in HCC treatment.

## 2. Materials and Methods

### 2.1. The Human Proteome Map (HPM)

HPM (http://www.humanproteomemap.org/index.php accessed on 14 October 2021) presents a draft map of the human proteome using high resolution Fourier transformation mass spectrometry. In-depth proteomic profiling of 30 histologically normal human samples, including 17 adult tissues, 7 fetal tissues, and 6 purified primary hematopoietic cells in humans [15]. Here we analyzed the protein expression level of tyrosine-metabolizing enzymes (tyrosine aminotransferase (TAT), HPD, homogentisate 1,2-dioxygenase (HGD), GSTZ1, and FAH) in different tissues.

### 2.2. The Cancer Genome Atlas (TCGA)

The somatic mutation data and RNA expression data were obtained from The Cancer Genome Atlas (TCGA) cBioportal platform (https://www.cbioportal.org/ accessed on 14 October 2021) [16,17]. The mutation was identified and analyzed in the cBioportal platform.

### 2.3. Gene Expression Omnibus (GEO)

The expression microarray series containing HCC tumor and non-tumor samples were downloaded from the Gene Expression Omnibus (GEO, https://www.ncbi.nlm.nih.gov/geo/ accessed on 14 October 2021) datasets: GSE14520 [18] and GSE84402 [19].

### 2.4. Gene Set Enrichment Analysis (GSEA)

Gene set enrichment analysis was performed using GSEA 4.0.3 (http://software.broadinstitute.org/gsea/index.jsp accessed on 14 October 2021) in which the hallmark gene set “c5.go.v7.4.symbols.gmt” was adopted. For the grouping of patients, we ranked the specific gene expression in order from high to low, and divided the number of patients in half. The top-ranked patients were divided into the high-expression group, and the remaining patients were divided into the low-expression group. For tyrosine-metabolizing enzymes, we used the expression of HPD as a specific gene.

### 2.5. The Kaplan-Meier Plotter (KM Plotter)

The Kaplan-Meier Plotter (http://kmplot.com/analysis/index.php?p=background accessed on 14 October 2021) [20], which runs meta-analyses based on the discovery and validation of survival biomarkers, is an online tool for assessing the effect of genes on tumor survival based on the large databases GEO, EGA, and TCGA.

### 2.6. The Human Protein Atlas (HPA)

The expression of SLC27A5, HGD, GSTZ1 and FAH by immunohistochemistry (IHC) comes from the HPA database (https://www.proteinatlas.org/ accessed on 14 October 2021) [21].

### 2.7. Cell Culture and Stable Knockdown Cell Lines

The human hepatoma cell line HepG2 and human embryonic kidney cell line HEK293T were grown in DMEM containing 10% (*v*/*v*) fetal bovine serum (FBS, ExCell Bio, China) and 1% (*v*/*v*) penicillin/streptomycin. All cells were cultured at 37 °C in an incubator supplied with 5% CO_2_. For SLC27A5 knockdown cell lines, lentivirus was generated by co-transfecting HEK293T cells with pLKO.1-shSLC27A5, envelop plasmid pMD2.G, and packaging plasmid psPAX2 using a polyethylenimine (PEI) transfection reagent (Polysciences, Warrington, PA, USA), following the manufacturer’s protocol. HepG2 cells were then infected with filtered lentiviral supernatant and selected with 2 μg/mL of puromycin.

### 2.8. RNA Extraction and Real-Time PCR Analysis (qPCR)

Trizol (Invitrogen, Carlsbad, CA, USA) was used to extract RNA according to manufacturer’s protocol. Real-time PCR was carried out using the LightCycler 96 (Roche, Switzerland). The β-actin gene was used as the reference gene to normalize the expression level between samples, and sample was calculated using the cycle threshold (2^−ΔΔCT^) method. All primer sequences for the specific and reference genes are listed in Table 1.

### 2.9. Reagent

Insecticide Diazinon (Catalog No. T0998) was purchased from TargetMol.

### 2.10. Statistics

Data were analyzed using GraphPad Prism 5 (GraphPad Software Inc., San Diego, CA, USA). All data are presented as mean ± standard deviation. Comparison of two groups was conducted using the two-tailed Student’s *t*-test. A value of *p <* 0.05 was considered to indicate a statistically significant difference.

## 3. Results

### 3.1. Tyrosine Metabolism Pathway Was Inhibited in HCC

**Figure 1:**
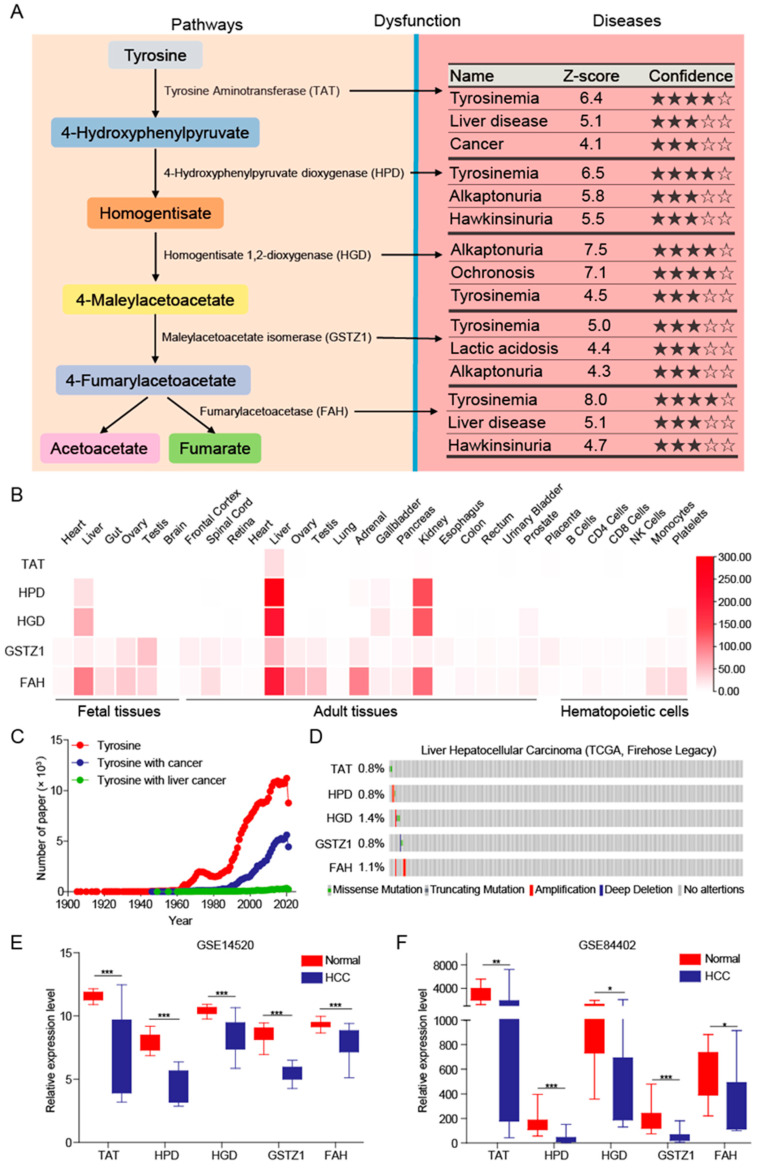
The tyrosine metabolic pathway is inhibited in HCC. (**A**) Tyrosine metabolism pathway and related diseases. (**B**) The expression level of tyrosine-metabolizing enzymes in different tissues. (**C**) Statistics on the number of articles published by different keywords. (**D**) The types and frequency of mutations of tyrosine-metabolizing enzymes in HCC patients. (**E**,**F**) The tyrosine-metabolizing enzymes’ mRNA expression levels between tumor and normal tissues in patients with HCC in GEO database. The *p* values were determined by the two-tailed *t*-tests (* *p* < 0.05, ** *p* < 0.01, and *** *p* < 0.001).

The tyrosine metabolism pathway plays an important role in biological processes and is related to the synthesis of neurotransmitters, hormones, and melanin. The dysfunction of the tyrosine metabolism pathway is related to a variety of diseases, including tyrosinemia, liver disease, and cancer [22,23,24] (Figure 1A). The final products of this metabolic pathway, fumarate and acetoacetate, are further converted into acetyl-CoA, which is involved in the biosynthesis of lipids. This implies that there is a crosstalk between tyrosine and lipid metabolism mediated by metabolites.

As the largest physical organ of the human body, the liver is an important place for material metabolism, especially lipid metabolism. Based on the HPM platform, we found that tyrosine-metabolizing enzymes are specifically and highly expressed in liver tissues, whether in fetal tissues or adult tissues (Figure 1B), which suggests an important role of tyrosine metabolism in the liver. Next, we analyzed the number of articles published on the NCBI and found that the number of articles related to tyrosine is huge, and there are also many articles related to tyrosine and cancer, but the number of articles about tyrosine in HCC amounts to few (Figure 1C). This shows that the underlying role and mechanism of tyrosine metabolism in liver has not been clearly elucidated.

In order to clarify the role and mechanism of tyrosine-metabolizing enzymes in HCC, we first analyzed the mutation frequency of related proteins, and found that there were not many mutations and no obvious characteristics, which showed that mutations did not affect HCC (Figure 1D). Next, we downloaded datasets which contained HCC and adjacent normal liver tissue from the GEO database. We found that the expression of tyrosine aminotransferase (TAT), HPD, homogentisate 1,2-dioxygenase (HGD), GSTZ1, and FAH at mRNA level was significantly decreased in HCC (Figure 1E,F). The expressions of HPD and GSTZ1 were found to be significantly down-regulated in the HCC patients collected in the reported articles [4,25]. Taken together, these findings suggest that the tyrosine metabolism pathway is significantly inhibited in HCC and may play a crucial role in HCC development.

### 3.2. Reduced Tyrosine Metabolism Predicts Poor Prognosis in HCC Patients

**Figure 2:**
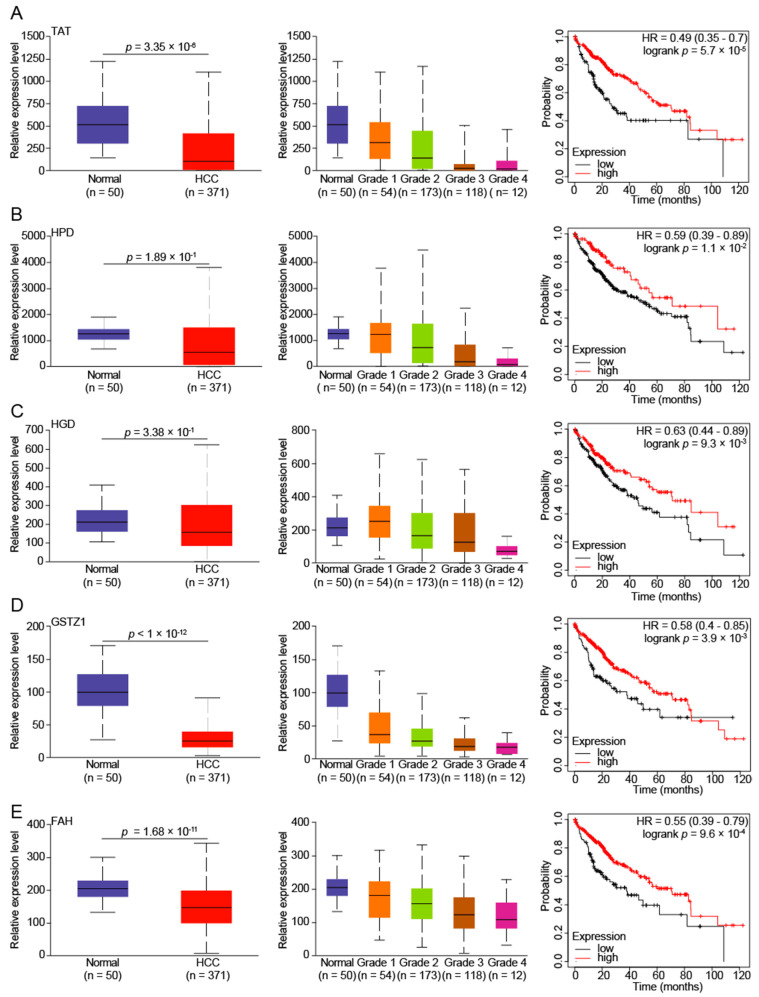
Low expression of tyrosine-metabolizing enzymes is associated with poor prognosis in HCC patients. The tyrosine-metabolizing enzymes ((**A**)-TAT, (**B**)-HPD, (**C**)-HGD, (**D**)-GSTZ1, and (**E**)-FAH) mRNA expression levels between tumor (of different grades) and normal tissues in patients with HCC in TGCA database. Overall survival of patients with HCC grouped by tyrosine-metabolizing enzymes’ expression through the Kaplan-Meier Plotter online analysis tool.

It has been reported that amino acid metabolism is a new target for tumor therapy [1,26], and we found that the mRNA level of tyrosine-metabolizing enzymes in HCC was significantly down-regulated. To explore the significance of tyrosine-metabolizing enzymes in HCC, we analyzed the mRNA expression of tyrosine-metabolizing enzymes in the TCGA database and observed similar results with the GEO database (Figure 2). Furthermore, we also found that the expression of tyrosine-metabolizing enzymes gradually decreased as the grade of HCC increased (Figure 2). This suggests that the level of tyrosine metabolism indicates the degree of HCC progression. Lastly, the Kaplan-Meier analysis of tyrosine-metabolizing enzymes and overall survival (OS) in patients with HCC were performed using the Kaplan-Meier plotter online analysis tool. The results suggest that a low expression of tyrosine-metabolizing enzymes in tumor tissues are significantly associated with poor OS in patients with HCC (Figure 2). In short, low expression of tyrosine-metabolizing enzymes is associated with a poor prognosis in HCC.

### 3.3. Tyrosine Metabolism Regulates Cell Cycle

**Figure 3:**
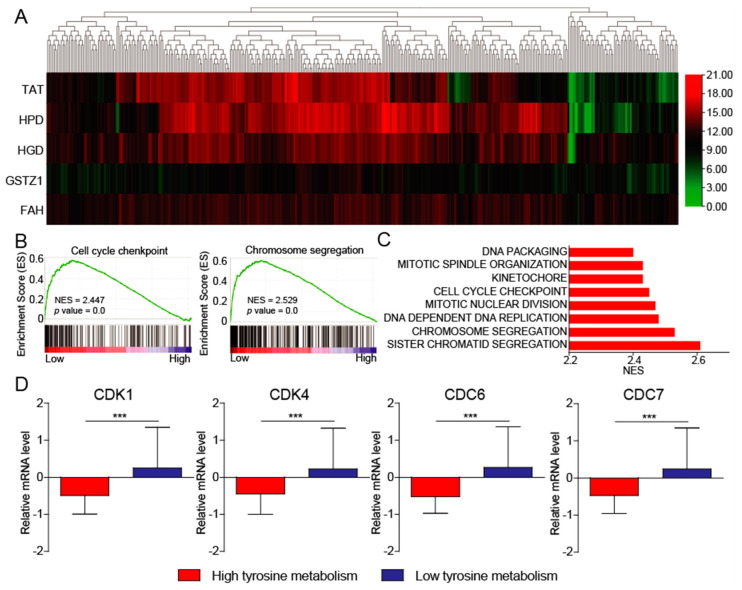
The tyrosine metabolism regulates the cell cycle. (**A**) Hierarchical clustering of five tyrosine-metabolizing enzymes transcript levels across HCC tissues in TCGA-LIHC datasets. (**B**) GSEA pathway enrichment analyses of five tyrosine-metabolizing enzymes’ signature in patients with HCC from the TCGA-LIHC datasets. (**C**) The core-enriched signaling pathways in high and low tyrosine metabolism groups. NES, normalized enrichment score. (**D**) The expression of cell cycle-related gene in high and low tyrosine metabolism groups. The *p* values were determined by the two-tailed *t*-tests. *** *p* < 0.001.

To uncover the mechanism of the low expression of tyrosine-metabolizing enzymes in regulating HCC development, we utilized the publicly available datasets (The Cancer Genome Atlas-Liver Hepatocellular Carcinoma (TCGA-LIHC)) for pathway enrichment analysis. First, we divided the patients into two groups with high and low tyrosine metabolism (Figure 3A). We next sought to investigate the altered signaling pathways driven by the decreasing of tyrosine metabolism in HCC. Gene set enrichment analysis (GSEA) of pathway enrichment showed that cell cycle checkpoint and chromosome segregation were significantly enriched in patients with HCC, with a low expression of tyrosine-metabolizing enzymes in the TCGA-LIHC datasets (Figure 3B). Both cell cycle checkpoints and chromosome separation are important events in cell cycle, which play a crucial role in HCC progression. Here, we also found that tyrosine metabolism may regulate HCC progression through affecting the cell cycle. Consistent with this result, DNA replication and packaging, as well as nuclear division, are also regulated by tyrosine metabolism (Figure 3C). In addition, we also found that the expression of important cell cycle regulatory genes (CDK1, CDK4, CDC6, and CDC7) was significantly increased in patients with low tyrosine metabolism (Figure 3D). Moreover, it has been previously reported that GSTZ1 is involved in the regulation of the cell cycle in HCC [27]. All in all, these results indicate that low-level tyrosine metabolism leads to the abnormal activation of the cell cycle and promotes the progression of HCC.

### 3.4. The SLC27A5 Is Positively Correlated with Expression of Tyrosine-metabolizing Enzymes and Regulates Cell Cycle

**Figure 4:**
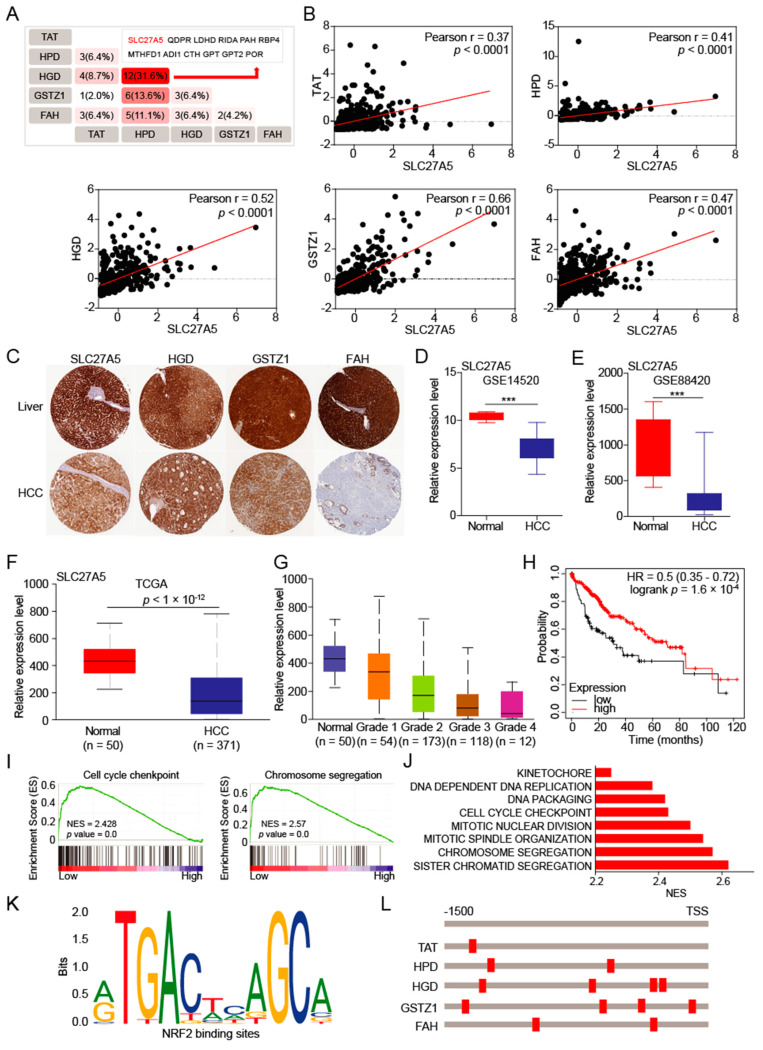
SLC27A5 regulates the expression of tyrosine-metabolizing enzymes. (**A**,**B**) The overlapping analysis for co-expressed genes of five tyrosine-metabolizing enzymes in HCC patients. (**C**) Representative IHC staining of SLC27A5, HGD, GSTZ1 and FAH in HCC from HPA database. (**D**–**F**) The SLC27A5 mRNA expression levels between tumor and normal tissues in patients with HCC in the GEO and TCGA database. (**G**) The SLC27A5 mRNA expression levels between different grades of tumor and normal tissues in patients with HCC in TGCA database. (**H**) Overall survival of patients with HCC grouped by SLC27A5 expression through the Kaplan-Meier Plotter online analysis tool. (**I**) GSEA pathway enrichment analyses of SLC27A5 expression signature in patients with HCC from the TCGA-LIHC datasets. (**J**) The core-enriched signaling pathways in high SLC27A5 level and low level groups. (**K**,**L**) The binding motif of transcription factor NRF2 and tyrosine-metabolizing enzyme promoter. The *p* values were determined by the two-tailed *t*-tests. *** *p* < 0.001.

To determine the upstream regulator of tyrosine-metabolizing enzymes, we comprehensively analyzed the co-expressed proteins of five tyrosine-metabolizing enzymes in HCC. The results showed that the SLC27A5 protein may be a key candidate regulatory protein (Figure 4A). We further analyzed and found that SLC27A5 expression has a significant positive correlation with tyrosine-metabolizing enzymes in HCC patients (Figure 4B). The results from HPA database also showed that SLC27A5, HGD, GSTZ1, and FAH are repressed in HCC tissues, and expression of TAT and HPD was not detected (Figure 4C). Overall, tyrosine metabolism may be regulated by SLC27A5.

Next, we found that SLC27A5 was also significantly decreased in HCC patient tumors from the GEO and TCGA datasets (Figure 4D–F). Similarly, the expression of SLC27A5 decreases as the grade of HCC increases (Figure 4G), which shows that the expression of SLC27A5 affects the grade of HCC. And low expression of SLC27A5 in tumor tissues was significantly associated with poor OS in patients with HCC (Figure 4H). When we divided HCC patients into SLC27A5 high expression and low expression groups, we found that the low expression group, like the low expression group of tyrosine metabolism, also activate the cell cycle (Figure 4I,J). These results indicate that SLC27A5 and tyrosine-metabolizing enzymes are important regulators of cell cycle and HCC progression.

The nuclear factor erythroid 2-related factor 2 (NRF2) is a common underlying link between lipid synthesis and oxidative stress, which plays an indispensable role in metabolic homeostasis by regulating oxidative responses [28]. Gao et.al found that SLC27A5 regulates antioxidant gene via the KEAP1/NRF2 pathway in HCC [29]. To determine whether SLC27A5 regulates the expression of tyrosine-metabolizing enzymes via the KEAP1/NRF2 pathway, we found that there are NRF2 binding elements on the promoters of the five metabolic enzymes (Figure 4K,L). This suggests that tyrosine-metabolizing enzymes are likely to be transcriptionally regulated by the transcription factor NRF2.

### 3.5. KnockdownSLC27A5 Promotes Growth of Human Hepatoma Cell

**Figure 5:**
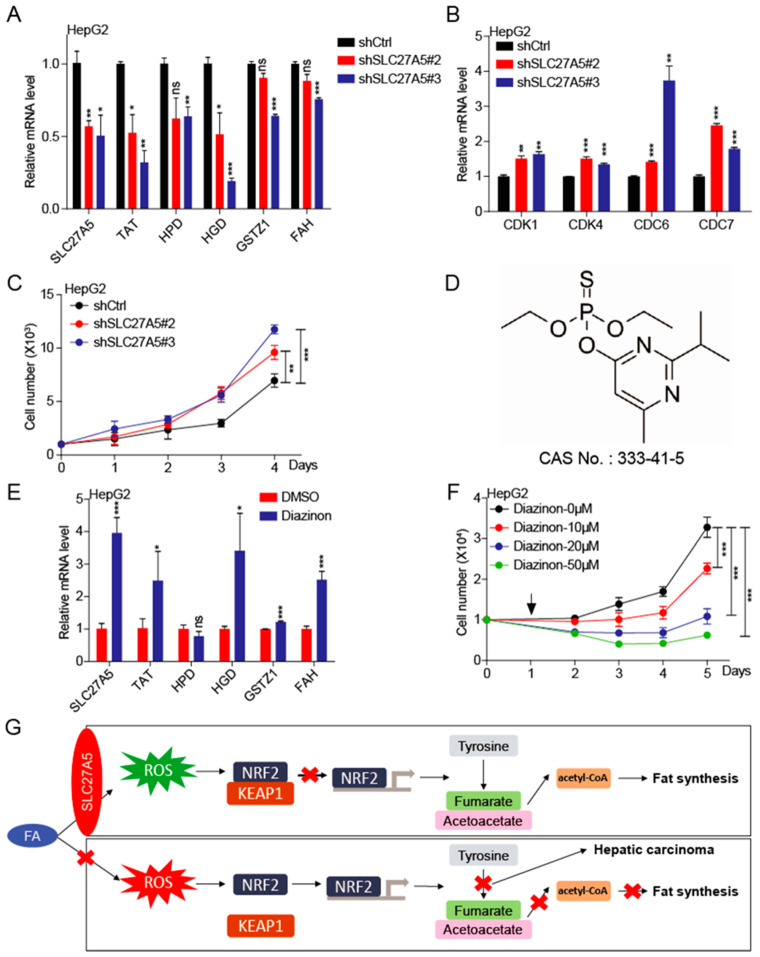
Knockdown SLC27A5 promotes growth of HCC cells. (**A**,**B**) The expression of SLC27A5, TAT, HPD, HGD, GSTZ1, FAH, CDK1, CDK4, CDC6, and CDC7 in HepG2-shCtrl and shSLC27A5 cells by qPCR. (**C**) The growth curve of HepG2-shCtrl and shSLC27A5 cells. (**D**) The chemical structure and CAS number of diazinon. (**E**) The expression of SLC27A5, TAT, HPD, HGD, GSTZ1, and FAH in HepG2 cells with the treatment of diazinon by qPCR. (**F**) The growth curve of HepG2 cells with the treatment of diazinon. The arrow represents the treatment of diazinon. (**G**) Proposed model: The role and mechanism of SLC27A5 in HCC progression. The *p* values were determined by the two-tailed *t*-tests. * *p* < 0.05, ** *p* < 0.01, and *** *p* < 0.001.

In order to further verify that SLC27A5 regulates the progression of HCC, we established HepG2-shSLC27A5 cell lines and found that knockdown SLC27A5 suppressed the expression of tyrosine-metabolizing enzymes (TAT, HPD, HGD, GSTZ1, and FAH) (Figure 5A). Meanwhile, the important cell-cycle regulators (CDK1, CDK4, CDC6, and CDC7) significantly increased in knockdown SLC27A5 cells (Figure 5B). These results suggest that the expression of tyrosine-metabolizing enzymes is regulated by SLC27A5 and ultimately affects the cell cycle. In line with our previous analysis results, knockdown SLC27A5 promotes the growth of HCC cells when compared with the control group (Figure 5C). The above results confirm our hypothesis: SLC27A5 regulates the expression of tyrosine-metabolic enzymes and the cell cycle.

Diazinon is a broad-spectrum insecticide extensively used to control pests in crops and animals (Figure 5D). Studies have shown that diazinon produces tissue toxicity, especially to the liver [30,31]. In addition, diazinon not only induces an increase in ROS [32,33], but also causes metabolic disturbance [34]. We found that diazinon not only induces an increase in the expression of SLC27A5, but also promotes the up-regulation of the expression of tyrosine-metabolizing enzymes (Figure 5E). At the same time, diazinon significantly inhibited the growth of HepG2 cells (Figure 5F), which proves that targets of SLC27A5 to regulate the expression of tyrosine-metabolizing enzymes may be therapeutic targets of HCC.

## 4. Discussion

The dysfunction of tyrosine metabolic pathways will lead to tumors and genetic diseases. The study of Fu and Lei reported that the tyrosine-metabolizing enzymes TAT and GSTZ1 are both suppressors of HCC [27,35]. Consistent with previous research, we found that the metabolism level of tyrosine in HCC is significantly inhibited, and indicates a poor prognosis for the HCC patients. This shows that tyrosine metabolism plays a role in tumor suppressor. Further analysis found that the inhibition of tyrosine metabolism promotes the cell cycle, which may imply that HCC cells use tyrosine as raw materials for other metabolic pathways, for example, in the re-synthesis of protein, because cell division needs to synthesize proteins.

Through co-expression analysis, we identified that the SLC27A5 protein regulates the expression of tyrosine-metabolizing enzymes by the transcription factor NRF2, which in turn affects the process of tyrosine metabolism. The SLC27A5 protein is highly expressed in hepatocytes, responsible for transporting extracellular dissociating fatty acids, and then providing raw materials for lipid synthesis. Importantly, we found that the SLC27A5 protein is also significantly suppressed in HCC. This means that the liver of HCC patients have lost the ability to absorb food-derived fatty acids, and the synthesis of lipids will also be blocked. In addition, due to the reduction of intake of fatty acids, NRF2 enters the nucleus to inhibit the transcription level of tyrosine-metabolizing enzymes and to inhibit the tyrosine metabolism process. The final products of tyrosine metabolism are important sources of acetyl-CoA conversion, so the endogenous lipid synthesis pathway is also hindered. This also implies that SLC27A5 plays a key role in lipid synthesis, and HCC cells do not need to store too much energy, but need to convert substances into materials that help cells to divide and proliferate quickly (Figure 5G). In addition, we found that the insecticide diazinon increases the expression of SLC27A5, so diazinon has a potential anti-tumor effect on HCC. In short, HCC cells provide a powerful support for the protein and DNA synthesis required for cell division by reducing the SLC27A5 sacrifice in the lipid synthesis pathway.

Hepatocellular carcinoma is the most common form of all primary liver malignancies and represents the third leading cause of cancer-related death globally. However, the current therapeutic effect of HCC is not satisfactory, so it is urgent to explore the potential mechanism of HCC development. In our study, it was found that HCC cells abandon the lipid synthesis storage pathway and use it for cell division and proliferation. Therefore, a diet low in fat might be favorable for HCC patients as it slows down the progression of HCC. Meanwhile, this might also help explain the weight loss of HCC patients, because the nutrition intake is directed by HCC cells, which promotes the development of HCC.

The occurrence of tumors is often accompanied by abnormal metabolic processes [36]. Therefore, understanding the cross-talk of different metabolic pathways in tumor cells is also an effective way to explore the mechanism of tumor occurrence and development. Our study reveals the connection between lipid metabolism and amino acid metabolism regulates the cell cycle, which in turn affects the progression of HCC, and provides novel insights for future research on the connection between different metabolic processes in HCC.

## 5. Conclusions

In conclusion, this study reveals the importance of SLC27A5 in HCC progression by coordinating metabolism processes, providing a mechanistic link between lipid metabolism and amino acid metabolism. Our study identified SLC27A5 and tyrosine-metabolizing enzymes as potential prognostic markers for HCC patients, which warrants further validation in different clinical settings. These will provide help for the development of novel treatment strategies for HCC in the future.

## Figures and Tables

**Table 1:** The sequence of primers for Real-time PCR.

Gene	Sequence (5′→3′)
*SLC27A5*	Forward primer: CATGGCGTGACAGTGATCCT Reverse primer: CAGCCCGTAGTCCATTGCC
*TAT*	Forward primer: CTGGACTCGGGCAAATATAATGG Reverse primer: GTCCTTAGCTTCTAGGGGTGC
*HPD*	Forward primer: GGAGCCCTGGGTAGAGCAA Reverse primer: CAAGAATTGGCCGATGTAGTTCA
*HGD*	Forward primer: ATTTACACCGAGTTTGGCAAGA Reverse primer: GGTCTCCTCAAAGACATCTATGC
*GSTZ1*	Forward primer: CGGGCATATACTGTGTAGGAGA Reverse primer: GGGGTGAGATCCACCTTGAA
*FAH*	Forward primer: TCTGCCACGATGCACCTTC Reverse primer: CCTTGTCCCTGAACATGATTCC
*CDK1*	Forward primer: GGATGTGCTTATGCAGGATTCC Reverse primer: CATGTACTGACCAGGAGGGATAG
*CDK4*	Forward primer: ATGGCTACCTCTCGATATGAGC Reverse primer: CATTGGGGACTCTCACACTCT
*CDC6*	Forward primer: CCAGGCACAGGCTACAATCAG Reverse primer: AACAGGTTACGGTTTGGACATT
*CDC7*	Forward primer: AGTGCCTAACAGTGGCTGG Reverse primer: CACGGTGAACAATACCAAACTGA
*ACTIN*	Forward primer: GGAAATCGTGCGTGACAT Reverse primer: TGCCAATGGTGATGACCT

## Data Availability

All data will be made available upon reasonable request by emailing the corresponding author.
